# COL5A2 Promotes Proliferation and Invasion in Prostate Cancer and Is One of Seven Gleason-Related Genes That Predict Recurrence-Free Survival

**DOI:** 10.3389/fonc.2021.583083

**Published:** 2021-03-18

**Authors:** Xiaohan Ren, Xinglin Chen, Kai Fang, Xu Zhang, Xiyi Wei, Tongtong Zhang, Guangyao Li, Zhongwen Lu, Ninghong Song, Shangqian Wang, Chao Qin

**Affiliations:** ^1^ The State Key Lab of Reproductive, Department of Urology, The First Affiliated Hospital of Nanjing Medical University, Nanjing, China; ^2^ Department of Urology, Shanghai Pudong Hospital, Fudan University Pudong Medical Center, Shanghai, China

**Keywords:** prostate cancer, Gleason score, recurrence-free survival, WCGNA, Col5a2

## Abstract

Extensive research has revealed that the score derived from the Gleason grading system plays a pivotal role in predicting prostate cancer (PCa) progression. However, the underlying involvement of Gleason-related genes in PCa requires further investigation. This study aimed to identify Gleason-related genes with the potential to guide PCa therapy and future research. Differentially expressed genes (DEGs) were identified by comparing PCa tissues with high or low Gleason scores using the Gene Expression Omnibus (GEO) and the Cancer Genome Atlas (TCGA) databases. R v3.6.1, SPSS v23, and ImageJ software were used for all analyses. An effective recurrence-free survival (RFS) predictive model based on seven Gleason-related genes was established and validated (TCGA, AUC = 0.803; five years, AUC = 0.740; three years, AUC = 0.722; one year, AUC = 0.711; GSE46602, AUC = 0.766; five years, AUC = 0.808; three years, AUC = 0.723; one year, AUC = 0.656; GSE116918, AUC = 0.788; five years, AUC = 0.704; three years, AUC = 0.693; one year, AUC = 0.996). Calibration and nomogram plots were conducted. Weighted correlation network analysis (WGCNA) was used, and COL5A2 was selected for further analysis. The results from *in vitro* experiments demonstrated that COL5A2 was upregulated in PCa with high Gleason scores. The knockdown of COL5A2 inhibited cell proliferation and invasion in PC-3 and LNCaP cell lines. Meanwhile, COL5A2 displayed a strong association with immune infiltration, which might be an underlying immunotherapy target for PCa. We successfully established a robust RFS predictive model. The findings from this study indicated that COL5A2 could promote cell proliferation and invasion in PCa.

## Introduction

PCa is the most prominent malignant tumor occurring in Western men (1, 2). It causes 1 to 2% of all deaths in men, and the associated morbidity varies between countries primarily due to the different rates of prostate-specific antigen (PSA) screening (1, 2). Clinically, androgen deprivation therapy (ADT) has long been considered the standard treatment for advanced PCa due to its ability to block the interaction between androgen receptors (AR) and androgenic ligands (3). Unfortunately, although most patients initially respond to hormone therapy, they eventually relapse and progress to a fatal disease called castration-resistant prostate cancer (CRPC) (4). Moreover, in men treated with radical prostatectomy, about 25% experience a biochemical recurrence (BCR), which may increase the risk of metastasis and cancer-specific mortality (5).

The Gleason grading system evaluates cell morphology after prostate biopsy (6). The system was first described by Dr. Donald Gleason and has become the cornerstone of PCa treatment and control (6). The score consists of adding the two most common grading modes for the tumors, with a score ranging from 2 to 10. It was demonstrated that as the score increased, the cancer-specific mortality rate gradually increased (7). Based on data from 20,845 patients undergoing radical prostatectomy, Epstein et al. reported that a considerable difference existed in the postoperative recurrence rate between a Gleason high-risk group (4 + 3 and higher) and a low-risk group (3 + 4 and lower) (8). Furthermore, in a multivariable model constructed by Pierorazio et al. using 7,869 PCa patients, the Gleason score was regarded as the most valuable predictive factor for RFS (9). Therefore, the Gleason system plays a pivotal role in guiding treatment and predicting the survival of patients with PCa (10).

Recently, the rapidly developing, high-throughput platforms used to determine gene expression have been applied widely for molecular classification, prognosis prediction, and targeting new drug discoveries (11). The broad discipline of bioinformatics can be applied to capture, store, analyze, and interpret biological data utilizing specific algorithms and software. The purpose of our study was to identify Gleason-related genes that played a vital role in PCa and might be novel targets in cancer therapy and the inhibition of cancer progression. These genes could provide directions for future research. Here, we identified differentially expressed genes (DEGs) between different Gleason graded PCa tissues and established an RFS predictive model using seven Gleason-related genes. COL5A2 was selected using WGCNA for further analysis, including clinical and immune infiltration analyses. Experimental results suggested that COL5A2 is highly expressed in high Gleason score PCa tissues and could promote tumor proliferation and invasion. Thus, this molecule might be a valuable therapeutic target for PCa.

## Materials and Methods

### Data Acquisition

The expression profiles, as well as clinical and survival information for PCa and control patients (489 tumor tissues and 51 normal tissues) were downloaded from the TCGA database (https://portal.gdc.cancer.gov/; Cancer Genome Atlas Prostate Adenocarcinoma (TCGA-PRAD) data collection; primary site: prostate gland, program: TCGA, data category: transcriptome profiling and clinical, data type: Gene Expression Quantification and Clinical Supplement) on March 23, 2020. The transcriptome data were the “HTseq-FPKM” file and the clinical data were the “bcr xml” file. The chip data derived from PCa patients were obtained from GSE70768 which contained 125 tumor samples and 118 matched benign tissues. GSE46602 and GSE116918 were used for model validation. The “affy” package included in the R software was used to read the original CEL files from the GEO database. We divided the samples into two categories, a high-score Gleason group with Gleason scores greater than 8 and a low-score Gleason group with Gleason scores less than 7. The flowchart of our study is shown in **Figure S1**.

### Difference Analysis and PPI Network Construction

The DEGs between the high- and low-Gleason score groups were analyzed using the “limma” package and defined as Gleason-related genes. The threshold of analysis was set as |logFC (fold-change)|>1 and a P-value less than 0.05. Common differential genes were confirmed after intersection analysis (TCGA and GSE70768). A PPI network was constructed using the STRING (http://string-db.org; Search Tool for the Retrieval of Interacting Genes) database, which is an online biological database that could help to uncover critical regulatory genes (12). Cytoscape_v3.8.0 was used to visualize the PPI network. Cytohubba, a plug-in for Cytoscape, was used to analyze the top 20 nodes with the most interactions.

### Construction of a Predictive Model and Nomogram to Predict DFS

As mentioned above, Gleason grades correlate closely with PCa recurrence. Hence, based on the Gleason-related DEGs we identified, univariate cox analysis, LASSO regression, and multivariate cox analysis were performed sequentially to screen for RFS-related genes. The prognosis model was established with “risk scores = Σcoef*Exp(*genes*)”. Clinical factors and risk scores were chosen to establish a nomogram for DFS. The nomogram was evaluated using calibration plots and ROC curves.

### Gene Set Enrichment Analysis (GSEA)

GSEA was conducted between high-risk and low-risk PCa patients to study the biological characteristics of our model. Specifically, the model was set such that “collapse data set to gene symbols” was false; the number of marks was 1,000; the “permutation type” was phenotype; the “enrichment statistic” was weighted; FDR was less than 0.25, and a nominal P-value of less than 0.05 were the cut-off criteria. The Signal2Noise metric was used to rank the genes. The high-risk group was regarded as the experimental group, and the low-risk group was used as the reference group. The “c2.cp.kegg.v7.1.symbols.gmt” gene sets database was selected for enrichment analysis.

### Weighted Correlation Network Analysis

To identify the significant mRNAs associated with the Gleason grades for the PCa cases, we constructed a co-expression network using the WGCNA package. The goodSamplesGenes function was applied to check whether the DEmRNAs of the data matrix met the criteria and to eliminate any unqualified data. The pickSoftThreshold function was used to calculate the value of β (a soft threshold power parameter) to ensure a scale-free network. We constructed a tree diagram using hierarchical clustering and calculated the correlation between the module eigengenes (MEs) and the clinical traits used to screen the MEs related to the Gleason grades for the PCa cases.

### Immune Correlation Analysis

The ssGSEA package was used to quantify the content of immune cells in TCGA samples. The most important advantage of this package was the high degree of freedom allowed in the quantifying process. The information for marker genes in 24 immune cells was obtained from Bindea et al. (13).

### Cell Lines and qPCR

The tissues used for qPCR analysis were obtained from the First Affiliated Hospital of Nanjing Medical University, which included all primary PCa tissue. The detailed information is shown in **Table S1**. This study was approved by the Ethics Committee of the First Affiliated Hospital of Nanjing Medical University. All patients indicated their approval for the use of their clinical tissues for research purposes. A normal prostate epithelial cell line (RWPE-1) and human prostate cancer cell lines (PC-3, DU145, LNCaP, and 22RV1) were purchased from iCell (Shanghai, China). Total RNA was isolated using Trizol (Invitrogen, Groningen, Netherlands). PrimeScript RT Master Mix (Takara, Japan) was used for cDNA synthesis. qPCR was performed using an SYBR Green assay for the analysis of COL5A2 mRNA expression according to the manufacturer’s instructions (Applied Biosystems, Foster City, CA, USA). The primers used were as follows. COL5A2, forward: 5′-CCGGGTCTAGCTGGTGAAAG-3′; COL5A2, reverse: 5′-TCTCCTCTAGGTCCTAACGGG-3′; GAPDH, forward: 5′-AC CACAGTCCATGCCATCAC-3′; and GAPDH, reverse: 5′-TCCACCACCCTG TTGCTGTA-3′.

### Protein Extraction and Western Blots

Total proteins were extracted from human PCa tissue using Western and IP lysis buffer (Beyotime, P0013; Beijing, China). The protein concentrations were measured using the BCA reagent kit (Pierce, 23227). The proteins were separated using 8% to 12% gradient SDS-PAGE gels and blotted onto polyvinylidene fluoride (PVDF) membranes. The membranes were blocked in TBS containing 0.1% Tween-20 (TBST) and 5% skim milk powder for 1 h at room temperature (RT). The primary COL5A2 and GAPDH antibodies were diluted (1:300, AtaGenix, Wuhan, China and 1:2,000, AtaGenix, Wuhan), respectively, then incubated with the membranes for 2 h at RT. The biotinylated secondary antibodies (anti-rabbit or anti-mouse IgG (H+L), CST, USA) were incubated with the membranes for 2 h at RT.

### RNA Interference Studies

RNA interference of COL5A2 was accomplished using small interfering RNA (siRNA). PC-3 and LNCaP cells were transfected with control siRNA and siRNA-COL5A2 using Lipofectamine 3000 (Invitrogen). The target sequence used for siRNA against COL5A2 was 5′-TTTGATTCCTATGGAGCCTGG-3′. Western blots and qPCR were used to evaluate the efficiency of siRNA interference.

### MTT Assay

The cells were seeded into 96-well plates in triplicate at a concentration of 2×10^3^ cells per well and treated with 100 μl of 0.5 mg/ml sterile MTT for 4 h (37°C, 5% CO^2^, for 24 h, 48 h, and 72 h). After the appropriate incubation period, the medium was removed, and 150 μl of dimethyl sulphoxide was added. The cell viability was determined using the MTT assay.

### Clonogenic Assay

The cells were inoculated into 30-mm cell culture dishes containing 10% fetal bovine serum (FBS) and cultured for 14 days. The medium was changed every three days. The cells were fixed with 4% formaldehyde for 15 min and stained with 0.1% crystal violet for 20 min before counting the number of cells present.

### Wound-Healing Assay

Cells were seeded into six-well plates and cultured until they reached 90% confluence. Cell gaps were marked in the bottom of each well using a 10-µl tip set to indicate cell boundaries at 0 h. Wound healing was observed after 24 h. The migration rate was calculated as follows: migration rate = (initial wound area-specific day wound surface area)/initial wound area × 100%.

### Transwell Invasion Assays

The Transwell assay was carried out using 24-well Transwell chambers. A total of 2.5×10^5^ cells were added to each upper chamber using a serum-free medium. Each bottom chamber was filled with 500 μl of culture medium containing 20% FBS. After 24 h, the cells that had migrated to the lower surface of the membrane were stained with 0.1% crystal violet and photographed.

### Statistical Analysis

All analyses were performed using R version 3.6.1, SPSS version 23, and ImageJ software. All statistical tests were two-sided, and a P-value less than 0.05 was considered statistically significant. All experiments were repeated at least three times. An independent *t*-test was used to compare continuous variables that exhibited normal distributions. The Wilcox test was used to compare the continuous variables that were not normally distributed.

## Results

### Identification of Differentially Expressed Genes

**Figure 1:**
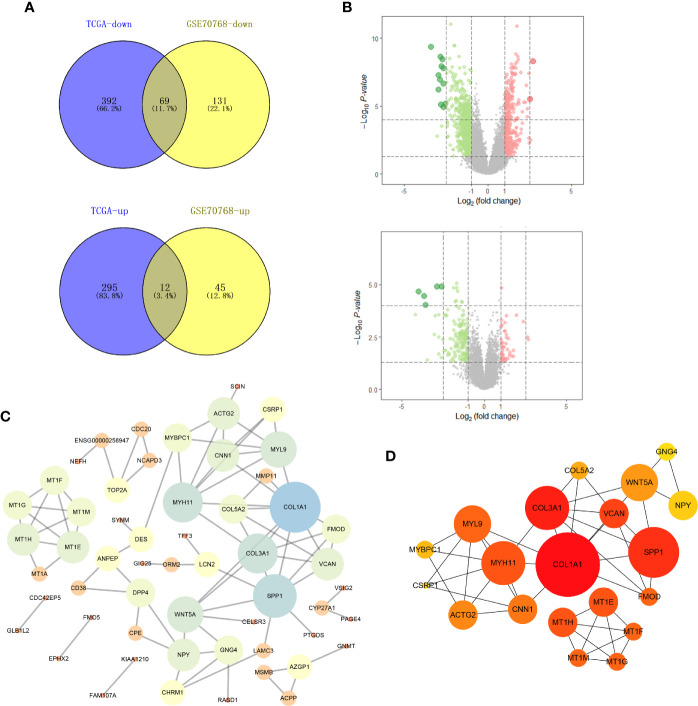
Identification of DEGs shared between the two databases and PPI network. **(A)** The Venn plot of TCGA-PRAD and GSE70768; **(B)** the volcano plot of TCGA and GSE70768; **(C)** PPI network of all DEGs; **(D)** top 20 nodes in PPI network. TCGA, The Cancer Genome Atlas; GEO, Gene Expression Omnibus; DEGs, differentially expressed genes; PPI, protein-protein interaction.

Genes that were differentially expressed between the high-risk and low-risk groups were analyzed using the limma package in R. In total, 768 genes were differentially expressed in the TCGA data, and 257 genes were differentially expressed in the GSE70768 data (**Figures 1A, B**). After completing the intersection analysis, 81 genes were related to Gleason scores, including 12 upregulated genes and 69 downregulated genes (**Table 1**).

**Table 1:** A total of 81 DEGs were identified from the TCGA and GEO datasets, with 12 upregulated and 69 downregulated.

DEGs	Gene names
Upregulated	COL5A2, MMP11, COL3A1, WNT5A, VCAN, TUBB3, LAMC3, TOP2A, CDC20, CELSR3, SPP1, COL1A1
Downregulated	MSMB, NPY, AZGP1, CDC42EP5, ANPEP, NEFH, MT1G, ACPP, MT1M, PAGE4, OR51E2, NCAPD3, PGC, SERPINA3, MT1F, ALOX15B, VSIG2, FOSB, ACTG2, TFF3, MYH11, KIAA1324, PTGDS, DES, CPE, CYP27A1, CHRM1, LCN2, PCP4, MYBPC1, ORM2, FAM107A, CNN1, MT1A, RLN1, GNG4, RASD1, GLB1L2, MYL9, EPHX2, KRT15, BCAS1, STXBP6, CSRP1, HMGCS2, KIAA1210, GMPR, SCIN, PCA3, CD38, POTEG, PRDM8, FMOD, FAM46B, DPP4, APOF, TGM3, GNMT, HSPB6, MT1E, TSPAN1, TCEAL2, RDH16, MT1H, TMEM158, FAM3B, FMO5, SYNM, AFF3

### Construction of the PPI Network

Based on the identified DEGs, we visualized the PPI network using the Cytoscape software (**Figure 1C**). According to the MCC value calculated by cytohubba, the top 20 crucial nodes were identified and are shown in **Figure 1D**.

### Establishment and Validation of the RFS Prediction Model

**Figure 2:**
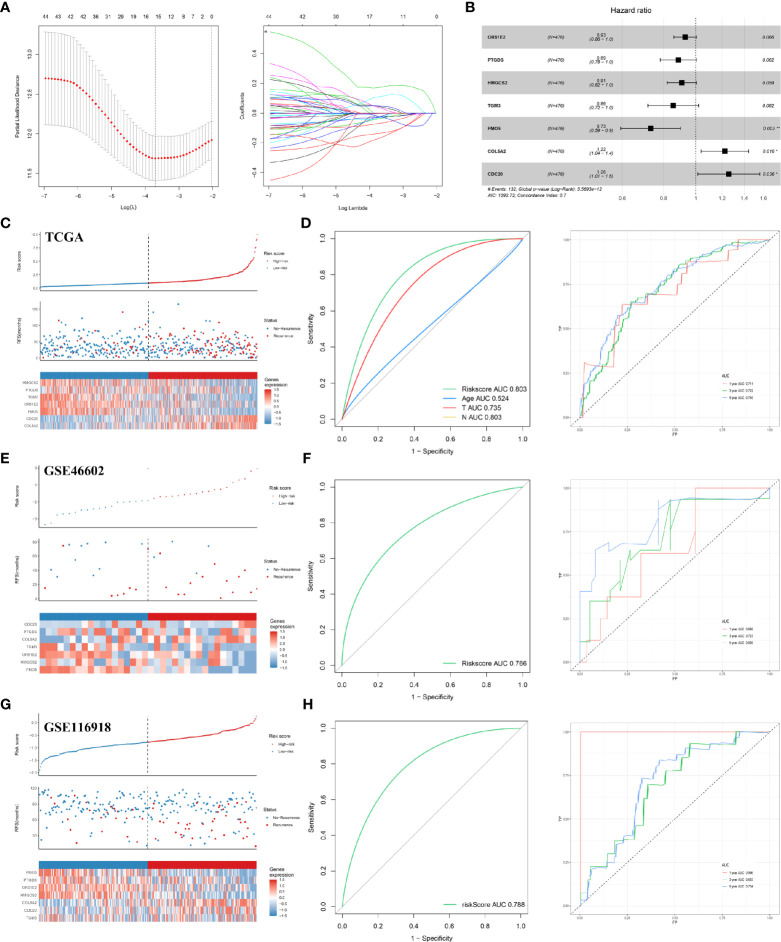
Construction and validation of the RFS predictive model. **(A)** LASSO coefficient profiles; **(B)** multivariate cox analysis of seven model genes; **(C)** the risk plot of RFS predictive model in TCGA; **(D)** ROC curve in TCGA; **(E)** the risk plot of RFS predictive model in GSE46602; **(F)** ROC curve in GSE46602; **(G)** the risk plot of RFS predictive model in GSE116918; **(H)** ROC curve in GSE116918. TCGA, The Cancer Genome Atlas; GEO, Gene Expression Omnibus.

We analyzed all the DEGs to identify a subset of prognoses that were statistically associated with the risk of recurrence, using univariate cox analysis, LASSO regression, and multivariate cox analysis (**Figure 2A**). Specifically, with P set to less than 0.05 as the screening criterion, 48 genes were associated with RFS after univariate cox analysis. The 48 genes were analyzed further using LASSO regression and multivariate cox analysis. With the LASSO regression analysis, the value of the optimal lambda was 0.0297. The results indicated that HMGCS2, PTGDS, TGM3, OR51E2, FMO5, CDC20, and COL5A2 were prominently associated with the risk of PCa recurrence (**Figure 2B**, **Table 2**). Based on these seven genes, a model for predicting postoperative recurrence of PCa was constructed using the formula, “OR51E2* -0.07+ PTGDS* -0.12+ HMGCS2* -0.10+ TGM3* -0.16+ FMO5* -0.31+ COL5A2* 0.20+ CDC20* 0.23”. Each patient received a risk score determined by the model. Based on the median value of the risk scores, we divided the patients into high-risk and low-risk groups (**Figure 2C**). The ROC curve revealed that our model exhibited good sensitivity and specificity in predicting PCa RFS (TCGA, AUC = 0.803; five years, AUC = 0.740; three years, AUC = 0.722; one year, AUC = 0.711) (**Figure 2D**). We also applied the model to the GEO datasets for validation. As expected, our model demonstrated satisfactory performance in the validation datasets GSE46602 (**Figures 2E, F**; AUC = 0.766; five years, AUC = 0.808; three years, AUC = 0.723; one year, AUC = 0.656) and GSE116918 (**Figures 2G, H**; AUC = 0.788; five years, AUC = 0.704; three years, AUC = 0.693; one year, AUC = 0.996).

**Table 2:** The seven genes of the RFS prediction model.

Genes	Coef	HR	HR.95L	HR.95H	P-value
OR51E2	-0.07	0.93	0.86	0.97	0.04
PTGDS	-0.12	0.89	0.78	0.96	0.03
HMGCS2	-0.10	0.90	0.82	0.98	0.04
TGM3	-0.16	0.86	0.71	0.96	0.04
FMO5	-0.31	0.73	0.60	0.89	0.00
COL5A2	0.20	1.22	1.03	1.44	0.01
CDC20	0.23	1.26	1.02	1.56	0.03

### Gene Set Enrichment Analysis (GSEA)

**Figure 3:**
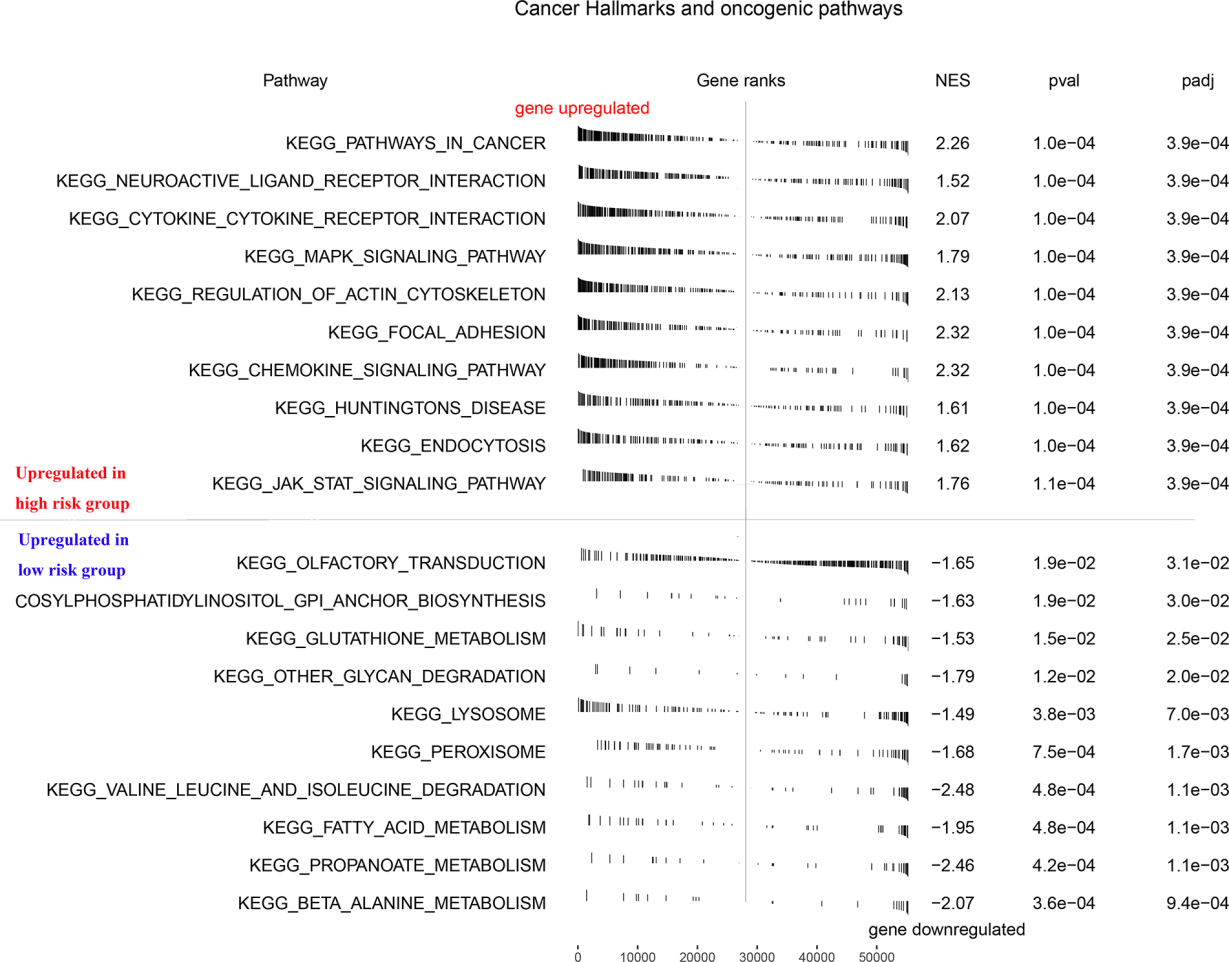
GSEA enrichment analysis of RFS predictive model. GSEA, Gene Set Enrichment Analysis.

To explore how the seven model genes described above were involved in the progression of PCa, we performed a GSEA based on the TCGA PCa cohort. As seen in **Figure 3**, the high-risk phenotype for the seven-gene model, the signaling pathways associated with cancer, neuroactive ligand-receptor interactions, cytokine-cytokine receptor interactions, the MAPK signaling pathway, regulation of actin cytoskeleton, focal adhesion, the JAK signaling pathway, and others were enriched (FDR < 0.25 and NOM P-value < 0.05).

### Nomogram and Calibration

**Figure 4:**
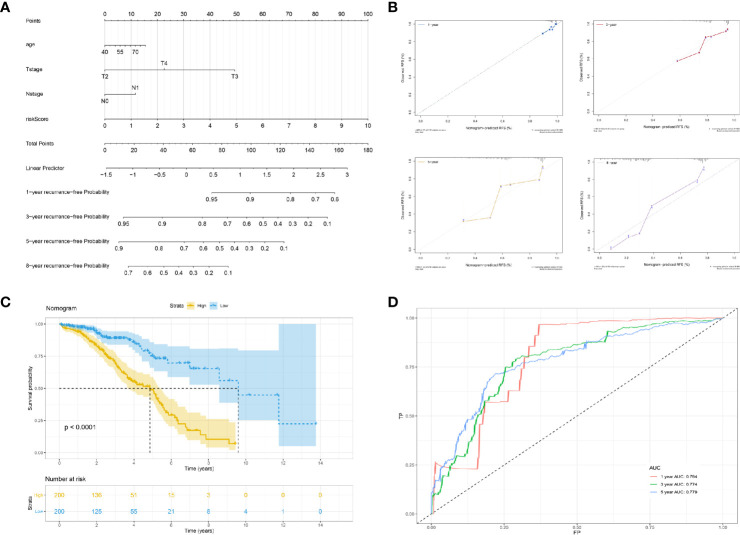
Construction of a nomogram based on risk score and clinical information. **(A)** The nomogram plot; **(B)** the calibrations of 1, 3, 5, and 8 years; **(C)** Kaplan-Meier curve of established nomogram; **(D)** ROC curve of established nomogram.

We constructed a multivariable cox proportional hazards model and plotted a nomogram based on the clinical factors and risk scores from the TCGA patients (the seven genes RFS prediction model). The primary clinical factors included age, T classification, and N classification (**Figure 4A**). The final point was the sum of the points from each item. As a continuous variable, the point of age was calculated as “0.3867* age - 15.465”. For the T classification, the patients in T2, T3, and T4 comprised 0 points, 49.398 points, and 22.535 points, respectively. N1 was 0 points, and N1 was 11.649 points. The point of our predictive model was “10* risk scores”. The one-year survival probability was “-2.15e-07* points ^3 + 3.1823e-05* points ^2 + -0.002542426* points + 1.049719044”. The three-year survival probability was “1.83e-07* points ^3 + -8.3045e-05* points ^2 + 0.002942615* points + 0.918472825”. The five-year survival probability was “4.12e-07 * points ^3 + -0.000115622 * points ^2 + 0.001462651 * points + 0.893415936”. The eight-year survival probability was “1.016e-06* points ^3 + -0.000155879* points ^2 + -0.001992433* points + 0.767177239”. The nomogram showed great effectiveness and stability when assessed using calibrations (**Figure 4B**), the Kaplan-Meier curve (**Figure 4C**; P<0.0001), and the ROC curve (**Figure 4D**; five years, AUC = 0.779; three years, AUC = 0.774; one year, AUC = 0.794). Compared with our predictive model, the nomogram, when combined with risk scores and clinical information, showed greater predictive effectiveness; for one year the AUC increased by 0.083 (Model AUC: 0.711, nomogram AUC: 0.789); for three years the AUC increased by 0.052 (Model AUC: 0.722, nomogram AUC: 0.774); and for five years the AUC increased by 0.039 (Model AUC: 0.740, nomogram AUC: 0.779).

### Weighted Gene Co-Expression Network Construction and Key Module Identification

**Figure 5:**
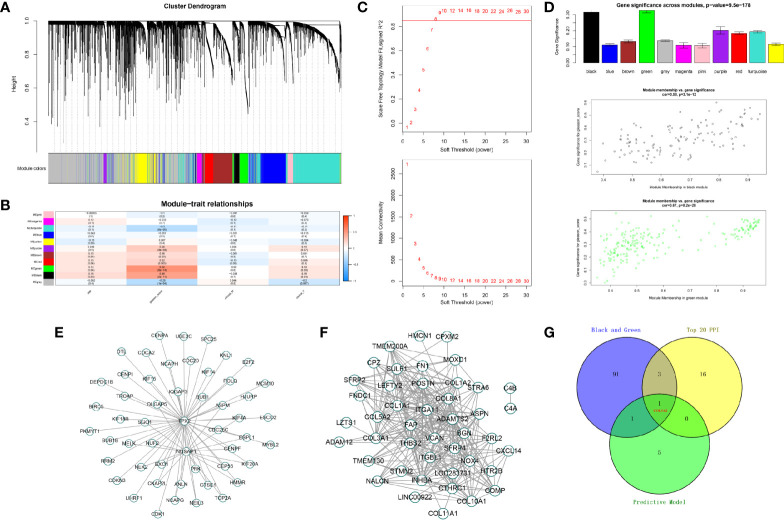
Identification of modules associated with the Gleason score in the TCGA-PRAD dataset. **(A)** The cluster dendrogram of co-expression network modules were ordered by a hierarchical clustering of genes based on the 1-TOM matrix. Each module was assigned to different colors; **(B)** module-trait relationships. Each row corresponds to a color module and column corresponds to a clinical trait. Each cell contains the corresponding correlation and P-value; **(C, D)** the green and black module (the corresponding correlation and P-value); **(E, F)** edges and nodes in green and black module; **(G)** The Venn plot of two module genes (black and green), seven model genes, and top 20 genes in PPI network of DEGs.

To identify protein-coding genes (PCGs) associated with Gleason scores in prostate cancer, we carried out a WGCNA analysis. In total, 489 tumor samples from PCa patients were clustered using the average linkage method and Pearson correlation analysis. We performed a network topology analysis of the various soft-thresholding powers to achieve relatively balanced scale independence and average connectivity of the WGCNA. In our study, the power of β=8 (scale-free R^2^) was selected as the soft-thresholding parameter to achieve a scale-free network. After merging modules with a dissimilarity less than 25%, 12 distinct PCG modules were identified (**Figures 5A–C**). Correlation analysis was performed between the MEs for each PCG module and the Gleason score. Subsequently, the black (Cor = 0.58, P <0.001) and green modules (Cor = 0.67, P <0.001) were identified as the PCG modules highly correlated with Gleason scores (**Figures 5D–F**). Finally, the intersection of two modular genes, the top 20 hub genes in PPI, and seven model genes identified only one gene, COL5A2, which was selected for further analysis (**Figure 5G**).

### Clinical Correlation and Immune Analysis

**Figure 6:**
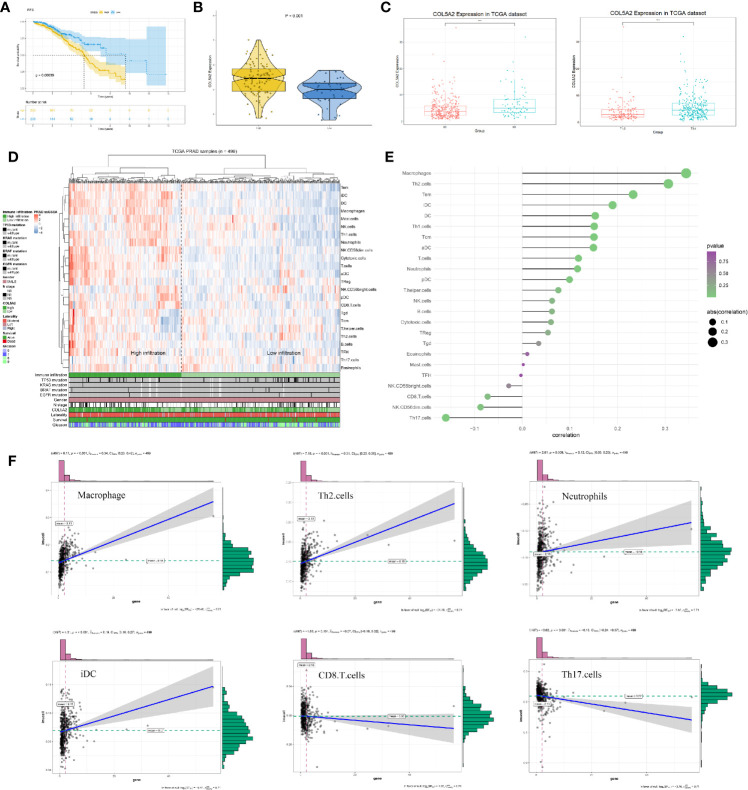
Clinical correlation and immune analysis of COL5A2. **(A)** Kaplan-Meier curve of RFS in high and low COL5A2 group; **(B)** the expression of COL5A2 in high and low Gleason score groups (GSE70768); **(C)** clinical correlation of COL5A2 in TCGA; **(D)** immune infiltration in TCGA-PRAD samples; **(E)** the association between COL5A2 and 24 immune cells; **(F)** the association between COL5A2 and some immune cells TCGA, The Cancer Genome Atlas.

Further analysis showed that COL5A2 correlated with poor RFS (TCGA) and was higher in PCas with high Gleason scores (GSE70768) (**Figures 6A, B**). The association between each subset of clinical information and COL5A2 was analyzed using R software and the Wilcox test. We found that the expression level of COL5A2 increased continuously in a stepwise manner in each subgroup of the T and N classifications (**Figure 6C**). Currently, immune function is more commonly reported to be related to PCa progression and the Gleason score (14). Therefore, we explored the underlying association between COL5A2 and immune infiltration of cells. The results indicated that the samples with high COL5A2 expression were correlated with high immune infiltration in the tumor microenvironment (**Figure 6D**). Further exploration of COL5A2 and multiple immune cell populations revealed that COL5A2 had a strong positive correlation with macrophages, Th2 cells, immature dendritic cells (iDCs), dendritic cells (DCs), and neutrophils, but a negative correlation with CD8 T cells, NK-CD56 bright cells, NK-CD56 dim cells, and Th17 cells (**Figures 6E, F**).

### COL5A2 Is Upregulated in PCas With High Gleason Scores

**Figure 7:**
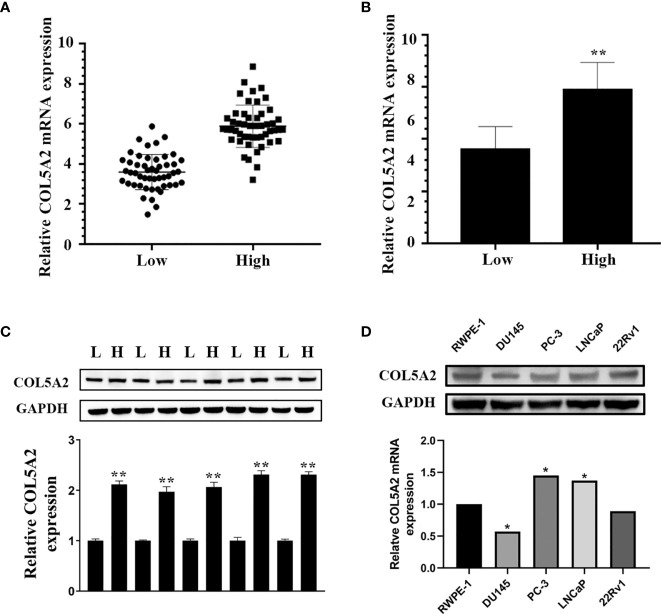
COL5A2 is upregulated in high Gleason score PCa. **(A, B)** Expression of COL5A2 was frequently upregulated in 50 high Gleason PCa samples compared with 50 low Gleason PCa samples by qPCR; **(C)** Western blotting of COL5A2 expression in high and low Gleason PCa samples; **(D)** qPCR analysis of COL5A2 expression in PCa cell lines. *P < 0.5; **P < 0.01.

We used qPCR to analyze the COL5A2 mRNA expression levels for 50 low-score Gleason PCa tissues and 50 high-score Gleason PCa tissues. We observed relatively high COL5A2 expression in the high-score Gleason group (**Figures 7A, B**). This result was also validated at the protein level using Western blots (**Figure 7C**).

### COL5A2 Promoted Proliferation and Invasion of PCa Cells

**Figure 8:**
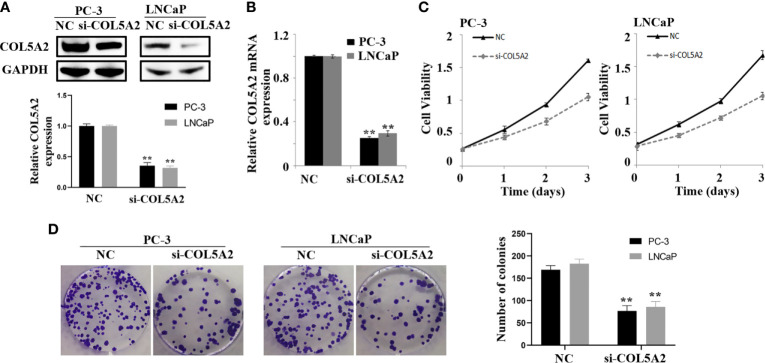
COL5A2 modulates the proliferation of PCa cells. **(A, B)** Western blotting and qPCR of indicated PCa cells transfected with COL5A2-RNAi-vector, COL5A2-RNAi; **(C)** MTT assays revealed that downregulation of endogenous COL5A2 significantly reduced the cell viability; **(D)** downregulation of endogenous COL5A2 reduced the mean colony number in the colony formation assay. **P < 0.01.

**Figure 9:**
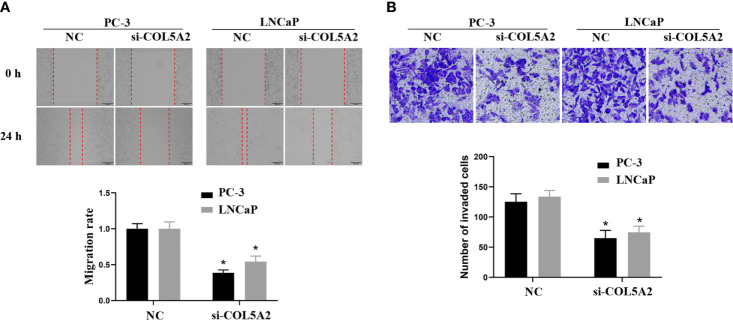
COL5A2 regulates the invasion of PCa cells. **(A)** Wound-healing assay revealed that downregulation of endogenous COL5A2 significantly reduced the migration rate. **(B)** Downregulation of endogenous COL5A2 reduced the number of invasion cells in the Transwell assay. *P < 0.05.

After the PCa cell lines were tested, a high expression of COL5A2 in mRNA and protein level was observed in PC-3 and LNCaP cells (**Figure 7D**). PC-3 and LNCaP cells were transfected with siRNA to knockdown the COL5A2 expression to validate the effects of COL5A2 on the PCa cells. Western blotting and qPCR revealed a successful knockdown of COL5A2 using siRNA interference (**Figures 8A, B**). The MTT assay indicated that knockdown of COL5A2 inhibited the proliferation of PCa cells (**Figure 8C**). The knockdown of COL5A2 also significantly decreased the number of colonies in the clonogenic assay (**Figure 8D**). The wound-healing assay revealed the presence of noticeably larger wound healing areas and a reduced rate of migration in si-COL5A2 cells compared with control cells (**Figure 9A**). Moreover, this result was validated by the Transwell assay in which the number of invading cells was significantly decreased in the si-COL5A2 group (**Figure 9B**).

## Discussion

In our study, we screened Gleason-related genes that were significantly associated with the recurrence of PCa in patients using a series of bioinformatics analyses. These genes may participate in cancer progression, and some have not been reported previously in PCa. Using WGCNA analysis, COL5A2 was identified for additional analysis and *in vitro* experimentation, and was shown to promote proliferation and invasion in PCa. The tissues from PCa patients and cancer cell lines were obtained from the First Affiliated Hospital of Nanjing Medical University.

The incidence rate for PCa has increased significantly in recent years (15). Although radiotherapy has achieved great success in treating advanced PCa, 10% to 45% of PCa patients are resistant to radiotherapy (16). The treatment strategy for PCa in the clinic is primarily based on the Gleason scores obtained from PCa biopsies. Pathologists specify the appropriate Gleason score after microscopic examination of PCa cellular morphology. Gleason scores provide one of the most robust prognostic indices for PCa patients and are a dominant influence factor for the BCR (17). Therefore, our study has provided considerable support to search for novel biomarkers associated with Gleason scores, find new therapeutic targets, develop new therapeutic methods, and provide new directions for future research.

Through the analysis of the GEO and TCGA datasets, 81 DEGs were found between the Gleason high-score group and the Gleason low-score group in PCa. Based on these DEGs, seven genes were identified to be associated with RFS. These genes were used to construct a model to predict the postoperative recurrence of PCa. KM and ROC curves revealed that the model was reliable for the training and validation groups. Furthermore, we observed that the high-risk group in our model was associated with several cancer pathways, including the MAPK signaling pathway, the JAK signaling pathway, and focal adhesion. Wang et al. reported that MAPK upregulation was associated with reduced survival in PCa. MAPK over-expression induced carcinogenic results through nonstandard activation of AKT/mTOR signals, while MAPK knockdown inhibited the proliferation of cancer cells (17). Also, by analyzing the data from 12 human PCa tissues and seven adjacent healthy tissues, Birnie et al. described the expression characteristics of 581 genes in PCa stem cells. They identified the JAK-STAT pathway and focal adhesion signaling as critical processes in cancer stem-cell biology (18).

Examination of the intersections for in-depth WGCNA analysis, model genes, and PPI hub genes demonstrated that COL5A2 was significantly correlated with Gleason scores. COL5A2 encodes an alpha chain for collagen type V, a type of fibro-regulated collagen that is upregulated in the stroma of different tumors (19). It has been reported that collagen type V is highly expressed in the matrix of pancreatic ductal adenocarcinoma and may affect the malignant phenotype of various pancreatic cancer cell lines by promoting adhesion, migration, and viability (20). Many studies have demonstrated that COL5A2 is differentially expressed in various tumors and is related to tumor metastasis and poor prognosis (21–23). However, up to now, the role of COL5A2 in PCa has not been examined. In our study, using *in vitro* experiments, we found that COL5A2 was upregulated in PCas with high Gleason scores and could promote the proliferation and invasion of PCa cells.

Considering the relationship between Gleason scores (and BCR) and immune function (14, 24, 25), we evaluated the effect of COL5A2 on immune infiltration. Our results revealed that COL5A2 remarkably increased the numbers of macrophages, Th2 cells, and DCs in the tumors. A recent study by Dang (2018) found that macrophages could stimulate cell proliferation in normal prostate epithelial cells, PZ-HPV-7, by secreting cytokines such as CCL3, IL-1ra, osteopontin, M-CSF1, and GDNF (26). The inflammatory response related to Th2 cells was also reported to have a conditional role in cancer (27). Additionally, DCs may play a critical role in tumor immune functions and have been tested for use in cancer vaccines (28). Simultaneously, the negative correlation of COL5A2 with CD8 T cells might result in a tumor-promoting effect in patients with high expression of COL5A2 (29). These results indicated that COL5A2 might have potential value for treatment and immunotherapy in PCa.

Our research presented several limitations. First, the amount of data published in the public database was limited. Thus, the clinical pathology parameters used for analysis in this study were not comprehensive and might lead to potential errors or biases. In subsequent studies, the number of samples will be increased to reduce bias and confirm the results. Second, all data series downloaded to construct the risk-scoring model were obtained from Western countries. Therefore, the results of this study may not apply to patients in Asian countries. Further research is needed to verify these results in Asian populations. Third, the mechanisms by which COL5A2 promoted the progression of prostate cancer requires further investigation.

## Conclusion

Using the information obtained from the serial bioinformatics analyses and *in vitro* experiments, we established a useful predictive model for RFS in PCa, based on seven Gleason-related genes. We also found that COL5A2 could promote proliferation and invasion in PCa, which had not been reported previously. Also, the interactions between COL5A2 and immune cells in the tumor microenvironment indicated that COL5A2 could be an underlying immunotherapy target in PCa.

## Data Availability Statement

The original contributions presented in the study are included in the article/**Supplementary Material** Further inquiries can be directed to the corresponding authors.

## Ethics Statement

The studies involving human participants were reviewed and approved by Ethics Committee of the First Affiliated Hospital of Nanjing Medical University. The patients/participants provided their written informed consent to participate in this study.

## Author Contributions

XR collected all the data and performed the bioinformatics analysis. XR and XW wrote the manuscript. KF, XC and XZ performed the experiment and statistical analysis. All authors contributed to the article and approved the submitted version.

## Funding

This work was supported by the National Natural Science Foundation of China (grant number 81972386).

## Conflict of Interest

The authors declare that the research was conducted in the absence of any commercial or financial relationships that could be construed as a potential conflict of interest.

## Supplementary Material

Supplementary Figure 1 The flowchart of our study.
