# Supplementary figures and images for: COL5A2 Promotes Proliferation and Invasion in Prostate Cancer and Is One of Seven Gleason-Related Genes That Predict Recurrence-Free Survival
Source: Front Oncol. 2021 Mar 18;11:583083. doi: 10.3389/fonc.2021.583083 (PMC8012814; doi:10.3389/fonc.2021.583083)

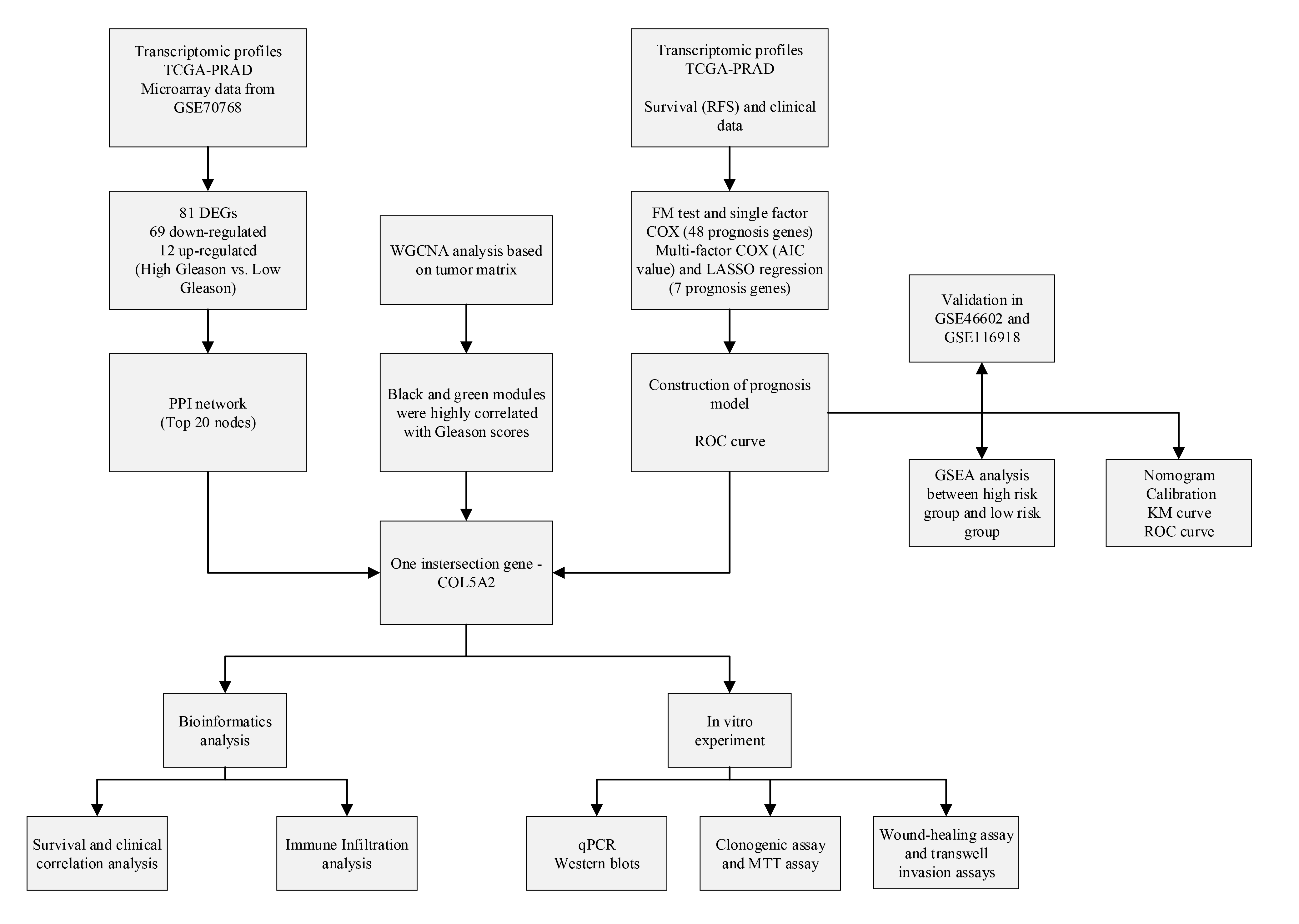

Supplement: Supplementary Figure 1 — The flowchart of our study. [file Image_1.tif]
