# Supplementary material for: COL5A2 Promotes Proliferation and Invasion in Prostate Cancer and Is One of Seven Gleason-Related Genes That Predict Recurrence-Free Survival
Source: Front Oncol. 2021 Mar 18;11:583083. doi: 10.3389/fonc.2021.583083 (PMC8012814; doi:10.3389/fonc.2021.583083)
Supplement: Supplementary file 2 [file Table_1.docx]

| **Table S1. Detailed information on 100 PCa patients.** | | |
| --- | --- | --- |
|  | Gleason high group (n= 50) | Gleason low group (n=50) |
| **T classification** |  |  |
| T1 | 11 | 13 |
| T2 | 15 | 18 |
| T3 | 21 | 17 |
| T4 | 2 | 0 |
| NA | 1 | 2 |
| **Age** |  |  |
| 30-45 | 2 | 0 |
| 45-55 | 7 | 5 |
| 55-60 | 11 | 15 |
| 60-70 | 27 | 28 |
| >70 | 3 | 2 |
